# Measuring 8 to 12 year old children’s self-report of power imbalance in relation to bullying: development of the Scale of Perceived Power Imbalance

**DOI:** 10.1186/s12889-019-7375-z

**Published:** 2019-08-05

**Authors:** Helen J. Nelson, Garth E. Kendall, Sharyn K. Burns, Kimberly A. Schonert-Reichl, Robert T. Kane

**Affiliations:** 1 School of Nursing, Midwifery and Paramedicine, Curtin University, Perth, Western Australia Australia; 2 School of Public Health and Collaboration for Evidence, Research and Impact in Public Health, Curtin University, Perth, Western Australia Australia; 3 Educational and Counselling Psychology, and Special Education, University of British Columbia, Vancouver, British Columbia Canada; 4 Human Early Learning Partnership, School of Population and Public Health, Faculty of Medicine, University of British Columbia, Vancouver, British Columbia Canada; 5 School of Psychology, Curtin University, Perth, Western Australia Australia

**Keywords:** Bullying, Power imabalance, Measurement, School health, Preadolescence

## Abstract

**Background:**

While power imbalance is now recognized as a key component of bullying, reliable and valid measurement instruments have yet to be developed. This research aimed to develop a self-report instrument that measures power imbalance as perceived by the victim of frequent aggressive behavior.

**Methods:**

A mixed methods approach was used (468 participants, Grade 4 to 6). This paper describes the exploratory (*n* = 111) and confirmatory factor analysis of the new instrument (*n* = 337), and assessment of reliablity and construct validity.

**Results:**

A 2-factor model represented *physical* and *social* aspects of power imbalance (*n* = 127: normed chi-square = 1.2, RMSEA = .04, CF1 = .993). The social factor included constructs of group and peer valued characteristics.

**Conclusions:**

This research will enhance health and education professionals understanding of power imbalance in bullying and will inform the design and evaluation of interventions to address bullying in children.

## Background

This paper discusses the development and validation of the Scale of Perceived Power Imbalance (SPPI), an instrument designed to measure children’s experience of power imbalance associated with bullying. The definition of bullying provides a basis for the development of the measurement tool [1]. For the purpose of this study, school bullying is defined as a form of aggression that is distinguished by *repeated* physical or emotional harm within a relationship of *power imbalance* [2]. This differs to the definition provided by Olweus [3], who included *intent* to harm in the definition of bullying. The criteria of intent differentiates purposeful acts of aggression from accidental harm [1]. It has been proposed that intentionality is understood within the context and goals of bullying: that harm is intended by the perpetrator, is perpetrated within the social dynamic of the peer group, and is perceived by the victim of bullying [4]. Based on a legal framework, it has been proposed that the judgement of intent rests on the likelihood that a reasonable person would foresee that aggressive behavior would result in harm [1, 5]. This criteria is difficult to apply as intent is not easily observed [1, 6]. For these combined reasons, the criteria of intent is not included in the definition of bullying for this paper.

The concept of repetition as an essential criteria of bullying has also been questioned by researchers, because single acts of aggression can provide an ongoing threat resulting in long term physical or emotional harm [5, 7]. However, the uniform definition of school bullying as unwanted aggression that is repeated and involves a power imbalance is accepted internationally [8, 9]. Consistent with this, repetition of aggression has been empirically associated with an experience of significantly greater threat and harm by targeted children than those who experienced aggression without repetition [10–12]. Children who are bullied perceive that they do not have the power to stop repeated aggression, contributing to an increase in harm [12]. The repeated acts of aggression that occur in a power imbalanced relationship place a load on neuroendocrine pathways that respond to stress, increasing the risk of poor health, learning and developmental outcomes [13]. Outcomes associated with bully victimization include fear, loss of hope, anxiety, depression and suicidal ideation [14]. The core concept that differentiates bullying from aggression is, therefore, the abuse of power by the perpetrator, and the experience of a power imbalance by the victim [4]. Despite the importance of the concept of power imbalance in bullying research, it is reported in recent literature that power imbalance has not been measured effectively, resulting in the inaccurate reporting of aggression as bullying [15].

Reports of bullying begin to increase in grade 4 to 6 (age 8 to 12) as children develop the cognitive capacity for self-reflection and place increasing value on social hierarchies [16, 17]. The concept of power imbalance as central to the definition of bullying has been driven by researchers, and children themselves may not consider repetition or power imbalance when talking about bullying [18]. They are, therefore, likely to report all acts of aggression as bullying [19]. With this in mind, researchers have sought to include the constructs of repetition and power imbalance in their self-report bullying instruments. Some researchers include a definition and ask children to consider the definition while answering questions [20]. There is evidence that many children fail to apply the definition of bullying correctly when answering the questions that follow and are prone to report aggressive behavior as bullying [15]. Other children may avoid answering questions truthfully when they read the word *bully* within the definition because of the stigma and shame associated with being a victim of bullying [21]. In this way the definition-based approach is contrary to conventional self-report measurement in psychology where questions ask about very specific behaviors and experience to discourage bias associated with respondents giving socially-desirable answers [22].

A second method of assessment seeks to increase the accuracy of measurement by asking children who report frequent victimization direct questions to assess perceived power imbalance. This is referred to as the behavioral-based method of assessment [23]. For example, Hunter et al. [10] aimed to differentiate bullying from victimization using three individual items to determine if the aggressor was physically stronger, in a bigger group, or more popular than the child completing the survey (ages 8–13). Concurrent and discriminant validity of the instrument were supported; students who experienced power imbalance perceived more threat and loss of control, and were at greater risk of depression. Similarly, the Californian Bully Victim Scale (CBVS) included three items to measure power imbalance from the perspective of the victim, asking “how popular, smart in schoolwork, and physically strong” the aggressor was [23]. Predictive validity was established; students who experienced power imbalance reported lower connectedness to school, life satisfaction, and hope. Five items were added to the CBVS: “how likeable, good looking, athletic, old, and how much money” the aggressor had in comparison to the victim [24]. These authors assessed the validity of a definition versus behavioral approach (grade 5 to 9) by comparing responses to one definition-based item from the Olweus Bullying Questionnaire (OBQ) [3]. Power imbalance was not significantly associated between the two measures. The authors concluded that the definition-based method might not detect some forms of power imbalance [24]. Notwithstanding, the accuracy of each item to detect the power differential using the behavioral approach remains unclear [25]. Cornell and Limber [26] claim that a satisfactory method to measure the power differential is yet to be identified.

Volk et al. [4] recommended that new instruments intended to measure bullying are based on insights gained from qualitative studies, and that specific forms of power are presented and validated. We have designed an instrument that uses this approach to measure the individual perception of power imbalance associated with repeated victimization at the level of the dyad. Our approach was innovative in that we worked with children aged 8 to 12 years to design an instrument and used factor analysis to explore the psychometric fit of items designed to measure the power imbalance component of bullying. The new instrument was implemented in an online survey.

The aim of this research was to establish the reliability and validity of the new instrument, named the Scale of Perceived Power Imbalance (SPPI). On the basis of previous work [27] we anticipated a negative association between perceived peer support and perceived experience of power imbalance by children who reported frequent victimization. We expected to find a moderate correlation of the SPPI with an existing measure of bully victimization, and that the SPPI would demonstrate reliability over time and invariance by gender, grade, and school.

## Methods

### Participants

Quantitative data collection occurred for Phase 1 in November 2015 and for Phase 2 in May and June 2016. Participants for each phase of the research were children in Grades 4 to 6 (aged 8 to 12 years) who were purposively sampled from low fee-paying private, independent schools in metropolitan Perth, Western Australia [28]. In Phase 1 participants were recruited from all eligible students (*N* = 174) via an information letter sent to parents from one school. Active consent was received for 121 students, of these 111 (64%) students were at school on the day of data collection and completed the online survey (59% female, 41% male). Thirteen percent of participants spoke a language other than English at home.

In the second phase of the research letters were sent to the principals of 10 primary schools inviting participation in the research. Of these, four principals agreed for their school to participate [29]. Participants were recruited from all eligible students (*N* = 642) via an information letter sent home by the school. Active consent was received for 351 participants, 14 students were absent on the day of data collection and one student did not assent to participate, therefore, a total of 337 (52%) students completed the online survey (51% male, 49% female). 24% of participants spoke a language other than English at home. The principal from one participating school (*N* = 174) was asked and agreed for data collection to include a retest after 2 weeks, active parental consent was received for 34% of students (*n* = 58), retesting was undertaken after 2 weeks, 50 of 58 participants (86%) completed the instrument a second time.

### Procedure

Ethics approval was obtained from the Curtin University Human Research Ethics Committee (RDHS-38-15) and the Principal of each participating school. Written informed consent was gained from the parents/guardians of the minors included in this study. Each school was informed in writing of the process, and was asked to arrange for immediate care by the school psychologist or chaplain should any child become upset as a result of answering the online questionnaire.

An online questionnaire was administered to participants in each school on one occasion in a classroom setting. On the day of data collection, the first author explained the research and the procedure to each group of students and answered any questions. Following this, students simultaneously completed the questionnaire. A research assistant was present to help children who had difficulty with reading and comprehension. A record of student questions was recorded in a research diary. A description of the measurement instruments included in the questionnaire is presented in the following section.

### Measures

#### Design of the new Scale of Perceived Power Imbalance (SPPI)

**Fig. 1:**
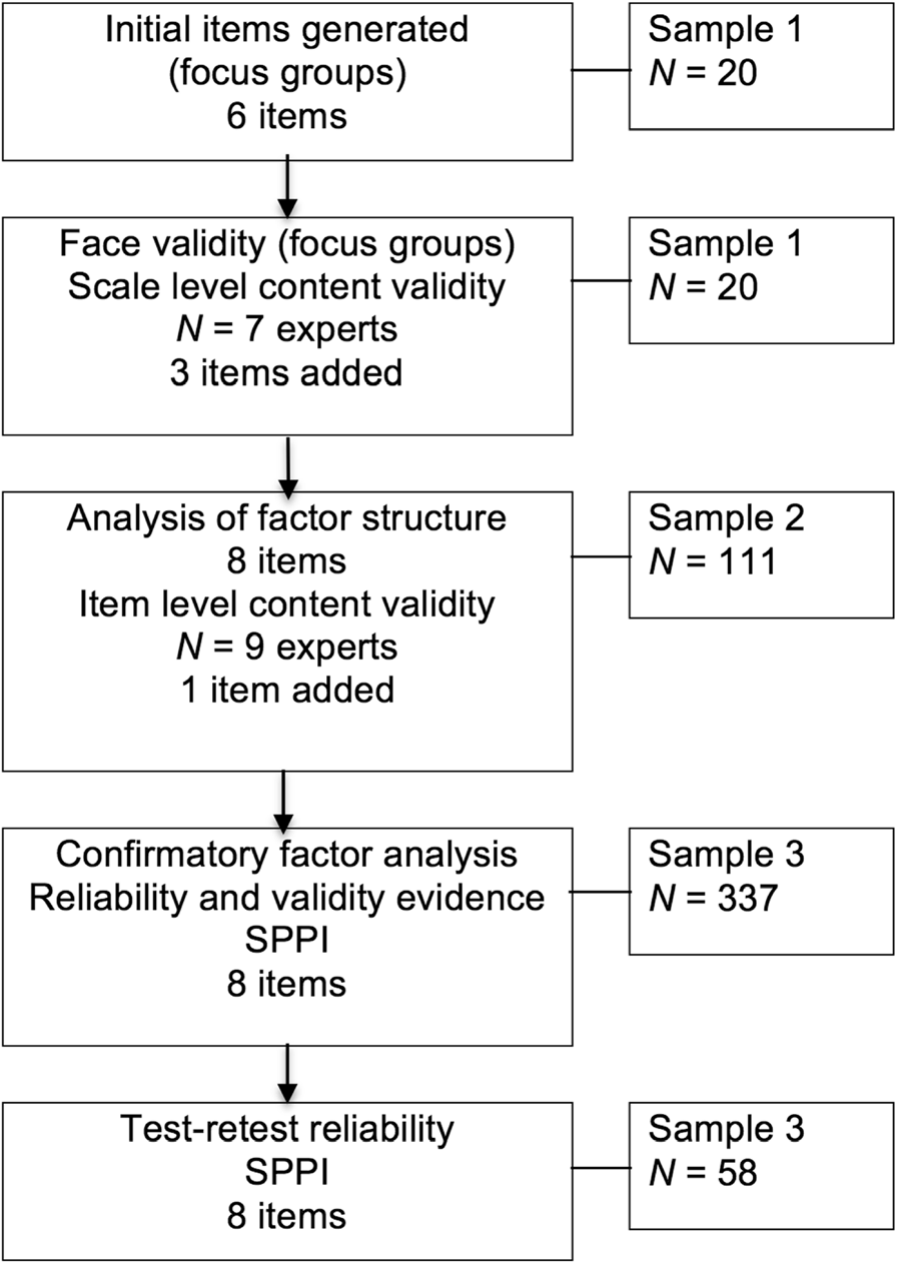
Stages of development of the new measure, *N* = number of participants, SPPI = Scale of Perceived Power Imbalance

The scale was developed for the local context using qualitative methodology [30]. The scale was displayed in response to children’ s report of frequent victimization and followed the stem question, “When these things happened to you was the mean student …” Six items were assessed for face validity by children, and for content validity by reviewers with expertise in education, psychology, social work, and public health (see Fig. 1 and Table 1). Items included “good looking” and “in the most popular group.” Universal agreement of scale-level content validity was given by the expert reviewers using the method recommended by Polit and Beck [31]. Two additional items were recommended by children who assessed face validity; “much stronger than you,” and “bigger than you,” and one additional item “with a group of students” was recommended at expert review (see Table 1). In Phase 1 the 9-item SPPI was displayed in response to children’s report of frequent aggression by the Adolescent Peer Relations Instrument (APRI) [32] or the Personal Experiences Checklist (PECK) [33] through the *display logic* function of Qualtrics™ online survey software. The binary response was 0 (*no*) or 1 (*yes*).

**Table 1:** Item development for the SPPI

	Sample 1 *n* = 20 Thematic analysis	Sample 1 *n* = 20 Face validity	Expert review *n* = 7	Sample 2 *n* = 111 Expert review *n* = 9	Sample 3 *n* = 337
Item 1	“Really smart”			“Really clever”	
Item 2	“Good looking”				
Item 3	“Older than you”				
Item 4	“Good at sport”				Item discarded
Item 5	“Trying to be more popular”				Item discarded
Item 6	“In the most popular group”				
Item 7		“Much stronger than you”			
Item 8		“Bigger than you”			
Item 9			“With a group of students”		
Item 10				“Tougher than you”	

Note: Table presents an outline the evolution of items included in the SPPI. Items 1 and 2 loaded onto the factor social power with acceptable factor loading (> .32), R^2^ however suggested 17.5% of the variance of Item 1 and 22.5% of the variance of Item 2 was explained by the social power factor

Exploratory factor analysis (EFA) (*N* = 111), revealed a 2-factor solution, 3-items represented *physical power* and 5-items represented *social power.* The data analytic approach and results of EFA are documented in the statistical analysis and results section. The item “really smart” was reworded to “really clever” because the factor loading was less than 0.5. An additional item “tougher than you” was added to form a 4-item subscale representing physical power (see Table 1). Expert reviewers (2 psychologists, 7 teachers) assessed the item content validity index (I-CVI) of the new instrument. The I-CVI of the four-item *physical power* factor was within the recommended range of .78 and 1.0 [31]. The I-CVI of three items of the *social power* factors were below the recommended minimum of .78: “good at sport” (.55) and “really clever,” “good looking” (.67). Items were based on theory and were designed with children, who are considered experiential experts and have a right to be heard [34]. This gave a priori evidence to keep the items unless repeated empirical testing found a statistically weak relation of the item to the factor [35].

In Phase 2 the SPPI was positioned in the online questionnaire following the APRI [32]. The SPPI was included twice in the questionnaire and followed a frequency of victimization of *2 or 3 times a month* as reported by the APRI; specifically the combined APRI verbal and physical victim scales (representing overt victimization), and the APRI social victim scale. The items of the APRI and the stem question are worded to capture the perceived goal of intent to harm, or feeling of hurt. This differentiates aggression from playful teasing.

#### Adolescent Peer Relations Instrument (APRI)

The APRI [32] was developed in Australia. Each six-item scale measures verbal, physical, and social victimization respectively and has demonstrated a clear factor structure and validity [36]. The APRI was reliable with primary school aged children (Grade 5, 6; *α* =.81 to .90) [37]. Following review of the instrument by children and an expert panel the wording of two items were adapted: “I was ridiculed” was changed to “students teased and made fun of me”, and “my property was damaged on purpose” was changed to “my property was hidden, taken or damaged on purpose.” A seventh item “a student said mean things behind my back” was added to the social victim scale to reflect language used by children in focus groups. The adapted 19-item APRI victimization instrument was answered using a six-point scale from 0 (*never*) to 5 (*every day*), responses of 4 and 5 were coded together (*several times a week/everyday*) to match the OBQ. Cronbach’s alpha scores were acceptable for each scale (.86 to .91) in the EFA research phase.

#### Personal Experiences Checklist (PECK)

The PECK was developed in Australia to measure children’s experience of being bullied by self-report (*n* = 647, age 8 to 15) [33]. The PECK did not define bullying and did not include a measure of repetition, intent, or power imbalance. The authors observed “it is arguable whether these elements are adequately assessed in any current measures of bullying” [33]. Items were simple to read and relevant to children of 8 years of age. Items included, “other students said mean things behind my back” and “other students teased me about things that aren’t true” [33]. Children who responded 0 (*sometimes*) to 4 (*most days*) to any item on the PECK verbal-relational scale answered the new power imbalance instrument in Phase 2. Cronbach’s alpha of the relational-verbal scale in this phase of the research was good (*α* = .94).

#### Olweus Bullying Questionnaire (OBQ)

The OBQ [3] is used to report prevalence rates of bullying victimization in schools. A definition of bullying precedes the item, “How often have you been bullied at school in the last few months?” The response is coded on a five-point scale: 0 (*I haven’t been bullied at school in the past couple of months*) to 4 (*Several times a week*). The screening question classifies non-involved victims of bullying quite accurately (specificity of 94.3%) but is not good at identifying true victims (sensitivity of 56.3%) [38]. Convergent validity of the new instrument was assessed with the OBQ screening question. The rates of bullying reported by multiple items are approximately double that of the single OBQ question [38]**.** The OBQ was placed last in the questionnaire so that children were not primed to think about bullying when answering behavior-based questions [39].

#### Perceptions of Peer Support Scale (PPSS)

The PPSS [40] measures children’s perception of friendships at school, and was included to measure the discriminant validity of the SPPI. Items are scored on a 3-point scale, 0 (*No*) to 2 (*A lot of the time*). A higher score indicates higher perceived peer support. The reliability of the PPSS with Grade 6 children in Western Australia was high (α = .92, *n* = 1163) [27]. Perceived peer support predicted the regular bullying (*p* < .05) and occasional bullying (*p* < .001) of other students. For example, children who reported that they bully others were more likely to answer that someone would chose them on their team “lots of times” than “sometimes” or “never” [27]. We therefore expected that children who report victimization with power imbalance would experience low levels of peer support. The PPSS was placed before the APRI in the online questionnaire.

### Statistical analysis

This paper reports the EFA and CFA that followed an extensive process of item development for the SPPI that emerged through qualitative research with children ages 9 to 11. The rationale for Phase 1 was to explore the factor structure of items identified through qualitative analysis [30]. The rationale for Phase 2 was that CFA would confirm a two-factor structure of perceived power imbalance, measuring *physical* and *social* characteristics of power. A minimum of five cases per variable was maintained throughout factor analysis [41].

#### Data analytic approach for exploratory factor analysis (EFA)

Data were assessed for frequency of responses to each item and missing data. EFA of the SPPI was run in SPSS using Principal Axis Factoring (PAF) with promax rotation. A Kaiser-Meyer-Olkin (KMO) value of .6 represented a minimum value for sampling adequacy [42]. Parallel analysis [43] was conducted, in conjunction with a scree plot, to determine the number of underlying dimensions [44]. Missing data were excluded listwise, and factor loadings less than .30 were suppressed [44].

EFA was continued in MPlus to confirm the factor structure identified in SPSS. EFA was based on the weighted least mean squares (WLSMV) estimator to account for the binary data and small sample sizes with non-normally distributed data [45]. Goodness of fit was reported with: Normed chi-square values < 3 [46]; comparative fit index (CFI > .90) [36]; Root-mean-square error of approximation (RMSEA < .08 [or a 90% CI that captures .08] indicates a reasonable fit; < .05 [or a 90% CI that captures .05] indicates a good fit); Standardised Root Mean Square Residual (SRMR ≤ .08) [47]. A minimum factor loading of .32 was accepted, .55 or higher was considered good [48]. The fit of items to relevant subscales was assessed and decisions made on initial items to be included [49]. Results were interpreted to give names to each subscale [50].

#### Data analytic approach of CFA

Data analysis began in SPSS; data were assessed for frequency of responses to each item and tolerance (values < .10 indicate multicollinearity) [51]. Analysis continued in MPlus Version 7 [52]. Missing data were deleted listwise, affecting 4.5 to 5.2% of responses for any one analysis. Fit indices as reported for Phase 1, and communality (R^2^ > .50) suggesting that 50% of the variance of the item can be explained by the factor to which it is linked [45]. Composite reliability of each factor was calculated [53]. Consistent with the method used by Marsh et al. [36] items were free to cross-load onto factors in the data set.

Second, we used multi-group analysis to determine how consistent the factor structure was over gender and grade. The maximum likelihood robust estimator (MLR) was used to account for incomplete and non-normal distribution of data [45]. Invariance of mean and covariance structures were assessed using the method detailed by Byrne [45]. Taking into account missing data, group sizes for the analysis were for gender: *girl, n =* 72*; boy, n =* 54, and for grade: *grade 4, n =* 50; *grades 5–6* (combined to account for smaller classroom sizes in some participating schools), *n =* 77. Invariance testing began with a configural model incorporating the baseline model for each group. There were no residual covariance’s to constrain equal in the configural model. Invariance analysis then tested for equivalence of factor loadings, covariance structures, intercepts, and latent factor means. The Satorra-Bentler Scaled Chi-Square [54] was used in Chi-square difference tests. Invariance was suggested by a non-significant corrected MLR chi-square value (*p* > .05) [45].

Third, test-retest reliability was assessed using Spearman’s rho (*r*_*s*_) to account for non-normal distribution of data. Test-retest reliability was calculated for the variable *bullied total* at Time 1 and Time 2. *Bullied total* represented self-report of frequent victimization and perceived power imbalance and was calculated by the combined ranked score of the 19-item APRI (0–4) and the SPPI (0 or 1): 0 (*Not bullied*) (APRI =0 or 1, Power = 0); 1 (*Frequent victimization without power imbalance*) (APRI = 2, 3, or 4, Power = 0); 2 (*2 or 3 times a month with power imbalance*) (APRI =2, Power = 1); 3 (*Once a week with power imbalance*) (APRI =3, Power = 1); 4 = (*Several times a week/Every day with power imbalance*) (APRI =4, Power = 1). Total score scales were whole numbers between 0 and 4, and matched the OBQ [3].

The final stage of analysis assessed the construct validity of the SPPI. Analysis began in MPlus allowing control for measurement error by assessing validity at the latent construct level. Discriminant validity was assessed by low correlations of a 3-factor model of social victimization (APRI), *social power*, and *physical power* (SPPI) in MPlus; and by negative correlations of a 3-factor model of children’s perception of peer support (PPSS; Ladd et al., 1996), *social power*, and *physical power* (SPPI) in MPlus. Convergent validity of the SPPI with the OBQ prevalence item was assessed in SPSS; Spearman’s rho (*r*_*s*_) was used to account for non-normal distribution of data.

## Results

### Exploratory factor analysis

The following number of children reported frequent victimization by each scale: APRI verbal (*n* = 48), APRI physical (*n* = 28), APRI social (*n* = 36), and PECK verbal-relational (*n* = 55). When missing data were deleted listwise, the number of participants who answered the power imbalance scale in response to the APRI verbal (*n* = 44)*,* physical (*n* = 28), and social scales (*n* = 34) was insufficient to run EFA, based on a minimum of five cases per item [41] or the minimum Kaiser criterion for sampling adequacy of .60 [42]. Results of the EFA are therefore reported on the power imbalance items that followed the PECK Verbal-relational scale (*n* = 51). Data were normally distributed. EFA in SPSS resulted in a KMO of .576 suggesting a “mediocre” fit of the data [55] and resulted in three eigenvalues ≥ 1. Because the eigenvalue criterion can lead to the extraction of too many factors [48] parallel analysis was calculated, resulting in a 2-factor structure. EFA of the 2-factor model in MPlus resulted in acceptable fit (*normed χ*^2^ = 1.25, RMSEA = .070 [90%CI = .00 to 0.148], CF1 = .942, SRMR = .119). The 90% confidence interval was wide; it included the value of .05 suggesting that the result reflected a small sample size. Factor loadings are shown in Table 2, the correlation between factors was −.039. Item 5 “trying to be more popular” did not load onto either factor.

**Table 2:** EFA of the power imbalance items in MPlus

Geomin Rotated Loadings of items			
	Factor 1	Factor 2	
Item 1	**0.491**	−0.285	Really smart?
Item 2	**0.905**	−0.125	Good looking?
Item 3	0.027	**0.556**	Older than you?
Item 4	**0.513**	0.193	Good at sport?
Item 5	0.200	0.139	Trying to be more popular?
Item 6	**0.764**	0.002	In the most popular group?
Item 7	0.013	**0.762**	Much stronger than you?
Item 8	−0.156	**0.902**	Bigger than you?
Item 9	**0.813**	0.271	With a group of students?

Note*:* bold = significant at 5% level

### Confirmatory factor analysis

CFA of the hypothesized 9-item 2-factor model of the SPPI did not provide a good fit to the data. On examination, the item “good at sport” had a low communality when answered in response to either *physical/verbal* or *social victimization* (*R*^2^ = .183, and .034 respectively), and did not load onto the factor when answered in response to the APRI social victim scale (standardized loading = .184). This was consistent with a high tolerance of the item (.919) indicating the uniqueness of the item. “Good at sport” was excluded from further analysis and an 8-item model of the SPPI was tested for fit.

Baseline model fit of the 8-item SPPI in response to *verbal/physical victimization* was acceptable (*n* = 146: normed *χ*^2^ =1.96, RMSEA = .081 [90%CI = .041 to .119], CF1 = .906). Standardized factor loadings ranged from .353 “really clever” to .941 “tougher than you”. The *R*^2^ for “really clever”, and “good looking” were low (see Table 3). In response to *social victimization* factor loadings of the baseline model of the 8-item SPPI were adequate (*n* = 127: normed *χ*^2^ =1.2, RMSEA = .04 [90% CI = .00 to .091], CF1 = .993). The communality of Items “really clever”, and “good looking” remained low (see Table 3). This is addressed in the discussion.

**Table 3:** CFA of the factor structures of power imbalance items in MPlus

Latent factor items	Overt victimization (*n* = 146)		Social victimization (*n* = 127)	
	Factor loading	R-square	Factor loading	R-square
Social power				
Really clever?	**0.353**	0.124	**0.419**	0.175
Good looking?	**0.388**	0.15	**0.473**	0.224
In the most popular group?	**0.894**	0.799	**0.790**	0.625
With a group of students?	**0.624**	0.389	**0.680**	0.462
Composite Reliability	*0.668*		*0.689*	
Physical power				
Older than you?	**0.475**	0.226	**0.646**	0.418
Much stronger than you?	**0.792**	0.627	**0.891**	0.793
Bigger than you?	**0.519**	0.27	**0.877**	0.769
Tougher than you?	**0.941**	0.885	**0.974**	0.949
Composite Reliability	*0.789*		*0.915*	

Note: **bold** = *p* significant at < 0.05. Power items were answered by children who reported frequent victimization by the APRI victim-verbal and victim-physical scales (Overt victimization), or by the APRI victim-social scale (Social victimization). Standardized factor loadings are reported

### Multi-group invariance

Multi-group analysis of the 8-item SPPI was conducted with the MLR estimator using the baseline models of best fit to assess invariance of the instrument. In each data set, the configural model of the SPPI answered in response to children’s self-report of *verbal/physical victimization* was inadmissible because of a negative residual variance for the item “in the most popular group” (gender,-.257; grade at school, −.043). The unstandardized factor loading for gender was high for *boy* (39.522, standardized =1.451), and for *Grade 5–6* (9.647, standardized =1.085).

Invariance was therefore assessed on the 8-item SPPI that was answered in response to self-report of social victimization. Invariance of the mean and covariance structures was demonstrated across gender as evidenced by a non-significant corrected *χ*^2^ between the nested model and the comparison model at each specified level of the model (*p* > .05). Invariance of the mean and covariance structures was likewise demonstrated between grade at school (Grade 4, and Grades 5–6) as evidenced by a non-significant corrected *χ*^2^ between the nested model and the comparison model at each specified level of the model (*p* > .05) (reported in Table 4).

**Table 4:** Multigroup analysis of the SPPI in response to social victimization

Model	*χ* ^2^	*df*	CFI	SRMR	No. F Parms	RMSEA	Invariance constraints	*χ*^2^ SCF	Model Comparison	CD	TRd	*df* diff	*p*
Power Social	Total group analysis (*n* = 127)						Factor1 by items 1, 2, 6, and 9; Factor 2 by items 3, 7, 8, and 10.						
CFA (*n* = 127)	23.152	19	.975	0.056	25	.041 [.00–.092]							
Invariance of covariance structures - gender													
CFA girl (*n* = 72)	23.659	19	.949	0.074	25	.058 [.00–.125]							
CFA boy (*n* = 54)	22.929	19	.945	0.076	25	.062 [.00–.140]							
MGGender-M1	46. 593	38	.947	0.075	50	.060 [.00–.112]	Configural	1.0608					
-M2	53.707	44	.941	0.077	44	.059 [.00–.108]	Factor loadings	1.0464	2 subtract 1	.9552	7.0740	6	*>.20*
-M3	54.086	47	.957	0.078	41	.049 [.00–.100]	Structural model	1.0430	3 subtract 2	.9931	.2142	3	*>.20*
Invariance of mean and covariance structures													
-M4	54.149	46	.950	0.079	42	.053 [.00–.103]	Factor loadings	1.0412	4 subtract 1	.9481	7.5848	8	*>.20*
-M5	61.099	54	.957	0.087	34	.046 [.00–.095]	Intercepts	1.0338	5 subtract 4	.9913	6.8441	8	*>.20*
-M6	59.467	50	.942	0.081	38	.055 [.00–.102]	Latent means	1.0269	6 subtract 4	.8625	4.8365	4	*>.20*
Invariance of covariance structures - grade													
CFA grade 4 (*n* = 50)	14.694	19	1.000	0.067	25	.000 [.00–.091]							
CFA grade 5_6 (*n* = 77)	21.438	19	.977	0.063	25	.041 [.00–.111]							
MGGrade-M1	36.274	38	1.000	0.065	50	.000 [.00–.082]	Configural	1.0653					
-M2	38.791	44	1.000	0.070	44	.000 [.00–.068]	Factor loadings	1.0497	2 subtract 1	.9509	2.1834	6	*>.20*
-M3	40.222	47	1.000	0.073	41	.000 [.00–.061]	Structural model	1.0312	3 subtract 2	.7599	.9976	3	*>.20*
Invariance of mean and covariance structures													
-M4	39.735	46	1.000	0.071	42	.000 [.00–.063]	Factor loadings	1.0288	4 subtract 1	.8554	2.6147	8	*>.20*
-M5	47.850	54	1.000	0.083	34	.000 [.00–.062]	Intercepts	1.0213	5 subtract 4	.9782	8.1681	8	*>.20*
-M6	44.132	50	1.000	0.077	38	.000 [.00–.064]	Latent means	1.0400	6 subtract 4	1.1688	4.2932	4	*>.20*

Note: *MG* Multiple group, *M1* model 1, *χ*^2^ indicates MLR chi-square value, *df* degrees of freedom, *CFI* confirmatory fit index, *TLI* Tucker-Lewis index, *No. FParms* Number of free parameters, *RMSEA* Root-mean-square error of approximation, *χ*^2^ *SCF* scaling correction factor, *CD* difference test scaling correction, *TRd* Satorra-Bentler scaled χ^2^ difference test, *df diff* difference in degrees of freedom between the nested model and the comparison model, *p* (significant at 0.05). M1 = Configural model, M2 = Factor loadings constrained equal, M3 = Factor variances constrained equal, M4 = Factor loadings constrained equal, M5 = Intercepts constrained equal, M6 = Latent means (factor means and variance estimated for group ‘boy’ only, ‘grade 5_6’ only). There were no residual covariance’s to constrain equal in the configural model

### Test-retest reliability

Test-retest reliability (2-week interval) of the combined ranked score (*n* = 50) from the APRI and SPPI was indicated by a moderately strong correlation when answered in response to self-report of *verbal/physical victimization r*_*s*_ =.773 (*p* < .001), and a strong correlation in response to *social victimization r*_*s*_ =.841 (*p* < .001).

### Validity

Construct validity of the SPPI was assessed in relation to the preceding APRI social victim scale. CFA of the 3-factor model of *social victimization*, *social power*, and *physical power* resulted in a good fit (*n* = 124: *normed χ*^2^ =1.09, RMSEA = .027 [90%CI = .00 to .057], CF1 = .983). Correlations between factors were low suggesting discriminant validity of the two latent factors of power imbalance (.294) and between the APRI social victim scale and power imbalance (*social power* = .325, *physical power* = .357).

Discriminant validity was supported by negative correlations between children’s perception of peer support (PPSS; Ladd et al., 1996) and each latent factor of the SPPI: *social victimization* (*physical power* = −.292*, social power* = −.175), CFA of the 3-factor model (*n* = 120: *normed χ*^2^ =1.0, RMSEA = .009 [90%CI = .00 to .041], CF1 = .997); and *verbal/physical victimization* (*physical power* = −.138, *social power* = .035), CFA (*n* = 140: *normed χ*^2^ =1.09, RMSEA = .025 [90%CI = .00 to .046], CF1 = .957).

Convergent validity was supported by a moderate correlation between the screening item of the OBQ [3] and the two latent factors of the eight-item SPPI (*overt* and *social victimization*) (*n* = 331, *r*_*s*_ =.533). Fifty five per cent of children reported victimization in a relationship of power imbalance by either the SPPI or the OBQ. The overlap in self-report of power imbalance between instruments was 20.2% (see Table 5). Fewer children reported being bullied by the OBQ screening item (23.8%) in comparison to those who reported frequent victimization by the combined ranked score of the 19-item APRI and the eight-item SPPI (50.8%) (see Table 5).

**Table 5:** Comparison of children’s self-report of victimization with power imbalance by instrument

	Frequency	Percent	Cumulative Percent
Did not report victimization with perceived power imbalance by either scale	149	45.0	45.0
Reported victimization with perceived power imbalance by combined rank score of APRI+SPPI only	103	31.1	76.1
Reported bully victimization by OBQ only	12	3.6	79.8
Reported victimization with power imbalance by APRI & SPPI and OBQ	67	20.2	100.0

Note: (*n* = 331). Fifty-five precent of children reported victimization in a relationship of power imbalance: 51.3% by the combined ranked scale of the APRI and SPPI, and 23.8% by the OBQ prevalence item. The overlap between instruments was 20.2%

An additional finding was that 11% of children who reported frequent social victimization and 8% of those who reported frequent overt victimization did not report an experience of power imbalance by the 8-item SPPI (see Table 6).

**Table 6:** Comparison of children’s self-report of frequent victimization with and without power imbalance

	No report of victimization (*n*)	Frequent victimization (*n*)	Frequent victimization with power (*n*)	Frequent victimization without power (*n*)	Percent of frequent victimization without power
Overt victimization (*n* = 335)	184	151	141	10	6.6%
Social victimization (*n* = 335)	202	133	119	14	10.5%

Note: Overt victimization = combined ranked score of the APRI victim-verbal, APRI victim-physical and the SPPI answered in response to self-report of overt victimization. Social victimization = combined ranked score of APRI victim-social and the SPPI answered in response to self-report of social victimization

## Discussion

Analyses of the items in the SPPI resulted in a two-factor structure. *Social power* measured the peer-valued characteristics of clever, appearance, athleticism, belonging to the popular group, and being with a group. *Physical power* measured the physical characteristics of age, size, strength, and toughness. Physical measures of power imbalance are more obvious, thus easier to quantify, than peer-valued characteristics, as evidenced by consistent fit to the data. Likewise, the group aspects of social power displayed consistently high factor loadings in response to children’s report of overt and physical victimization. The peer-valued characteristics of social power, “good at sport,” “clever,” and “good looking,” however, displayed inconsistent factor loadings and low communalities.

The factor *social power* measured characteristics that are valued by peers and associated with peer acceptance or belonging. The a priori factor structure was supported by the extant literature. The item “good at sport” did not load onto the social power factor in CFA. This is in contrast to a high response rate of *yes* for children answering this item in the SPPI (30% to *verbal/physical victimization* and 22% to *social victimization*). When Green et al. [24] previously measured power imbalance by individual items, children also responded most frequently to the item “more athletic” (39.3%). The reliability of the measure of power imbalance was however, not reported by the authors. The low factor loading for “good at sport” in our research does not mean that athleticism is not associated with power imbalance, the low communality and high tolerance (0.919) of the item suggested, however, that it was not related to the other items of social power [51].

Felix et al. [23] found that the item “smart in school work” was possibly not a good question to address power imbalance based on a small response rate to the item. Despite this the item was retained in the CBVS [24]. Similarly, in our research less than 20% of the variance of the item “really clever” was explained by the *social power* factor to which it was linked. Items such as “good at sport” and “clever”, and their opposites, might reflect an academic or ability-related form of power imbalance. This warrants further investigation. Moreover, in focus groups, children referred to smart as “getting their way out of trouble” by hiding the behavior from adults [30]. This social manipulation resulted in feelings of hopelessness and the inability to escape the situation by the victim, increasing the power of the perpetrator over the victim. This is consistent with recent qualitative research. Teachers may not recognize perpetration of aggression by popular students and may even place the responsibility for victimization on the targeted student [56]. Resilience is fostered when children receive social support. It is, however, possible that some children perceive that the teacher is supporting the child who is doing the bullying and is therefore not available as a source of social support [6]. This form of power is difficult to assess, and has been difficult to quantify, but must not be ignored because it is associated with poor health outcomes [14]. For this reason, we propose that further investigation into “smart” or “clever” as a form of power imbalance might inform the development of prevention strategies, including the promotion of resilience.

The item “good looking” was used as a measure of power in the revised CBVS [24] and was found to be associated with power in focus group analysis [30]. However, we found that only 22% of the variance of the item was explained by the *social power* factor to which it was linked. In focus groups, children referred to “looks”, but beyond appearance, *looks* also related to clothes, shoes, smart phones, and possessions. These are all consistent with the attribution of appearance to social power [57]. These items were kept because: a) they may be providing important information regarding the latent construct; and b) the amount of variance they contribute may be important, even if their factor loading was consistently low [58].

Cascardi et al. [2] have suggested that popularity is potentially a superficial feature of power imbalance. Our initial invariance analysis models were not identified for the SPPI items answered in response to the *verbal/ physical victimization* sub-scale of the APRI due to very high factor loadings on the item “in the most popular group”. These results suggest that boys were likely to experience a very high experience of power imbalance when the aggressor belonged to the popular group. Girls in Grades 5 and 6 were similarly more likely to experience a high power imbalance if the aggressor belonged to the most popular group. This is consistent with qualitative findings that popularity is a key influence on bullying within the group [59].

Preliminary support was found for the construct validity of the SPPI. Discriminant validity was supported by the strong 3-factor structure of the PPSS, SPPI *physical power* sub-scale, and *social power* sub-scale. As expected the correlation between perceived peer support and perceived power imbalance was very low, suggesting that children who felt supported by peers experienced lower rates of victimization with power imbalance. Consistent with previous research we found that double the number of children reported being bullied when they answered questions about individual types of bullying compared to the screening item of the OBQ [39].

### Limitations

This study used a single method of anonymous self-report and there was no verification of victimization by a different source, for example peers or teachers. There are, however, ethical considerations with peer nomination [20]. Agreement between the multiple approaches of quantitative analysis did support the 2-factor solution of power related to peer-valued characteristics and physical characteristics. However, thematic analysis of focus group discussion resulted in three forms of power imbalance reflecting physical characteristics (age), peer valued characteristics, and group membership and position [30]. Future research might benefit from exploring group and peer-valued characteristics of power as different constructs.

The SPPI was developed for the local context, however the qualitative phase of the study was reported in the context of prior international research [30]. There is a great need to reduce the harm of bullying in schools [60]. Victims of bullying experience a power imbalance that hinders their perceived ability to stop the repeated aggression. For this reason it is necessary to continue investigating the nature and measurement of power imbalance.

A limitation of this study is the focus on power imbalance associated with traditional forms of bullying and not cyberbullying. The form of power imbalance is likely to differ between traditional and cyberbullying [1]. Bauman et al. proposed that the imbalance of power associated with cyberbullying should not be assessed by self-report where possible because of the difficulty in inferring power imbalance from a subjective response to online communication [1]. In contrast, it is widely recommended that self-report will provide the most accurate measure of the power imbalance in traditional bullying [9, 20, 38]. This research focused on providing evidence on the reliability and validity of a specific measurement technique to attain children’s self-reported experience of power imbalance associated with traditional forms of bullying [25].

## Conclusion

There is reasonable agreement among researchers concerning the influence of physical factors, such as age, size, and toughness, on power imbalance. These have long been acknowledged in the literature and were found to have a strong factor structure in the analysis undertaken in this study. The influence of social factors, such as being good looking, smart, or popular, on power imbalance is much less clear for a number of reasons. Firstly, social forms of power are subtle in nature and not easily recognized by authority figures. Secondly, social power draws strength through peer group dynamics. This is supported in this study by the strong factor loadings of items that were specific to the peer-group. Thirdly, the factors that comprise social power are often highly valued by peers. The characteristics, themselves, are neutral; it is the status that peers attribute to these characteristics that confers power on those who possess them. Further research is required to better understand the influence of social factors on power imbalance. Specifically, we need to understand how social power reflects: 1) the dominance goals of the perpetrator; 2) group functioning; and 3) the perceived inability of the targeted child to overcome repeated victimization.

## Data Availability

The datasets used and/or analyzed during the current study are available from the first author on reasonable request.

## Abbreviations

APRI: Adolescent Peer Relations Instrument
CFA: Confirmatory factor analysis
CVBS: Californian Bully Victim Scale
EFA: Exploratory factor analysis
I-CVI: Item content validity index
OBQ: Olweus Bullying Questionnaire
PECK: Personal Experiences Checklist
PPSS: Perceptions of Peer Support Scale
SPPI: Scale of Perceived Power Imbalance
